# Validity of a single-item indicator of treatment side effect bother in a diverse sample of cancer patients

**DOI:** 10.1007/s00520-022-06802-3

**Published:** 2022-01-14

**Authors:** Pip Griffiths, John Devin Peipert, Andrea Leith, Alex Rider, Lucy Morgan, David Cella, Kim Cocks

**Affiliations:** 1 Adelphi Values, Bollington, UK; 2 Department of Medical Social Sciences, Northwestern University Feinberg School of Medicine, Chicago, IL USA; 3 Adelphi Real World, Bollington, UK

**Keywords:** Symptom bother, Psychometric validity, Health-related quality of life, Oncology, FACT-G, GP5

## Abstract

**Purpose:**

With higher efficacy of cancer therapies, the numbers and types of side effects experienced by patients have also increased, evidencing a need for brief assessments of side effect bother. The Functional Assessment of Cancer Therapy-General (FACT-G) includes the item “I am bothered by side effects of treatment” (GP5). This study aimed to confirm GP5’s validity in a large, diverse, real-world patient sample.

**Methods:**

Real-world data were drawn from 10 Adelphi Disease Specific Programmes (DSP™) conducted between 2015 and 2019 in France, Germany, Italy, Spain, the UK and the USA, covering 10 cancer sites. We examined correlations between GP5 responses and varied measures of patient-reported global health and the number of side effects experienced. We explored whether more advanced patients and those with worse Eastern Cooperative Oncology Group Performance Status Rating (ECOG PSR) reported greater side effect bother. Finally, we conducted differential item functioning (DIF) assessment using the Mantel–Haenszel approach.

**Results:**

The sample included 6755 advanced cancer patients. GP5 responses were distributed similarly across most cancer sites. A moderate, negative correlation (*r*_polyserial_ =  − 0.43) between GP5 responses and global health evidenced convergent validity. Known groups validity was evidenced by dichotomised distributions of GP5, showing expected results between cancer stage 2 vs. 3 and 4 and with ECOG PSR (*p* < 0.001). Little evidence of DIF was found.

**Conclusion:**

GP5 exhibited evidence of validity across cancer sites and countries and appeared to measure the same construct across these countries. GP5 has significant promise as a summary indicator of side effect bother.

## Introduction

There were nearly 4 million incident cases of cancer in Europe in 2018, and the incident rate remained high at over 400 cases per 100,000 people (age standardised rate [ASR]) [1]. Considering all cancer sites and both sexes, cancer accounted for approximately 2 million deaths in 2018. However, mortality due to cancer has decreased significantly for almost every cancer site [2]. For many cancer sites, the increase in survival rates is due, in part, to the greater efficacy of cancer therapy.

Despite the increased length of survival associated with effective cancer treatments, therapies also entail burdensome side effects and adverse events. Common side effects of cancer treatments include pain, fatigue, physical function impairment, sleep disturbance, gastrointestinal issues, itch and cognitive impairment and neuropathy [3–9]. Treatment side effects reduce health-related quality of life (HRQoL) among cancer survivors, especially during and directly after treatment [10, 11]. For this reason, patient-reported outcomes (PROs) measuring HRQoL, symptoms and side effects are often included as outcomes in clinical trials with the aim of determining which treatments impart the best experience around these outcomes.

The standard, clinician-driven assessment of side effects may not reflect the impact of the various side effects on patient HRQoL. A growing body of research argues that patients are in the best position to report on the level of both impact and tolerability associated with side effects [12–14]. Pearman et al. showed that the correlation between the raw number of clinician-rated side effects and a subjective measure of bother was low [15]. Directly asking patients how bothersome treatment-related side effects are should generate a measurement that relates more closely to the patient perspective of cancer treatment.

Assessment of the frequency and intensity of cancer treatment side effect bother must be efficient to be clinically feasible. They must also demonstrate validity to be useful. For these reasons, interest in a single-item summary measure of side effect bother has emerged. Specific attention has been paid to an item from the Functional Assessment of Cancer Therapy-General (FACT-G) GP5 item, which is rated on a five-point Likert scale from “Not at all” to “Very much” [16]. GP5 has evidenced validity: using multiple datasets compiled from clinical trials and prospective observational studies that included patients with multiple cancer sites; Pearman and colleagues found that (1) the mean GP5 response increased with adverse event (AE) toxicity grade; (2) the number of AEs experienced increased monotonically with worsening responses to GP5; and (3) HRQoL, as measured by the EuroQol-5 dimension (EQ-5D), decreased monotonically with worsening responses to GP5 [15]. For this reason, GP5 has already been employed in multiple trials evaluating cancer treatments [17–19].

Despite its use in clinical trials, GP5’s performance in real-world settings, especially in Europe, remains untested. Real-world data (RWD) collected in clinical settings can supplement clinical trials and help address some issues around trials ecological validity, which tend to emphasise internal validity over external validity [20]. RWD may better reveal actual clinical practice instead of a controlled environment with more selective inclusion and exclusion criteria for study participants. For this reason, there is increasing interest in RWD for drug evaluation, including detection of treatment side effects [21, 22]. This study aimed to assess the psychometric properties of GP5 in France, Germany, Italy, Spain and the UK; data from the USA were also included as a comparator. Specifically, we intended to assess (1) the item distribution of GP5 between cancer sites and countries; (2) the convergent and known groups validity of GP5; and (3) whether differential item function on GP5 exists between cancer sites and countries.

## Methods

### Participants and dataset

RWD were collected through Adelphi Disease Specific Programmes (DSP™). DSPs™ are large, multinational, independent point-in-time surveys conducted in clinical practice that describe current disease management, disease burden impact and associated treatment effects (clinical and physician-perceived) in a real-world setting. The DSP™ methodology has been previously published and validated [23–25]. Data for this study was drawn from 10 DSPs™ of advanced cancer patients covering multiple cancer sites with data collection between 2015 and 2019. As this data was from a real-world source, the number of patients with cancer at each site varied. Cancer sites were selected for inclusion in the study when there were ≥ 100 GP5 responses. This number was selected based on having a reasonable number of subjects for psychometric analysis. This led to 10 cancer sites: advanced breast, chronic lymphocytic leukaemia (CLL), diffuse large B cell lymphoma (DLBCL), follicular lymphoma (FL), gastric, hepatocellular carcinoma (HCC), melanoma, multiple myeloma (MM), non-small-cell lung carcinoma (NSCLC) and prostate cancer (PC). Patients were from European countries (the UK, France, Germany, Italy and Spain) and the USA.

Physicians completed a patient record form (PRF) for individual consecutively consulting patients who met the eligibility criteria. Physicians invited these same patients to voluntarily complete a patient self-completion form that included PROs. Physicians were identified by local fieldwork teams. To be eligible for inclusion, physicians had to be personally responsible for prescribing decisions and meet criteria specific to each disease area including year of qualification and number of patients seen per week or month. Patients were eligible if they met the following criteria:

- Diagnosed with the tumour type of interest
-  ≥ 18 years of age
- Receiving drug treatment for their cancer at the time of data capture
- Not enrolled in a clinical trial at the time of data capture

### Measures

The FACT-G item GP5 (“I am bothered by side effects of treatment”) was the focus of this study. As part of the Functional Assessment of Chronic Illness Therapy (FACIT) family of measures, the FACT-G is appropriate for use with any cancer type. The FACT-G has 4 subscales: physical well-being (PWB) (7 items, e.g. “I have pain”), social well-being (SWB) (7 items, e.g. “I get support from my friends”), emotional well-being (EWB) (6 items, e.g. “I worry about dying”) and functional well-being (FWB) (7 items, e.g. “My work (include work at home) is fulfilling”). The subscale scores are summed to create a total FACT-G scale score ranging from 0 to 108. GP5 is included in the PWB subscale [16]. At the start of the FACT-G, respondents are asked to indicate the response as it applies to the past 7 days. For GP5, like all FACT-G items, the response options are “Not at all”, “A little bit”, “Somewhat”, “Quite a bit” and “Very much”. For several tests, we dichotomised GP5 as “Not at all” or “A little bit” vs. “Somewhat”, “Quite a bit” or “Very much”, based on a sensitivity analysis. In the current study, participants completed GP5 as part of entire FACT-G in their native language [16].

In addition to the FACT-G, we collected the physician rated ECOG PSR [26]. ECOG PSR categorises patients into the following mutually exclusive functional groups: 0, “Fully active, able to carry on all pre-disease performance without restriction”; 1, “Restricted in physically strenuous activity but ambulatory and able to carry out work of a light or sedentary nature, e.g. light house work, office work”; 2, “Ambulatory and capable of all self-care but unable to carry out any work activities, up and about more than 50% of waking hours”; 3, “Capable of only limited self-care, confined to bed or chair more than 50% of waking hours”; 4, “Completely disabled, cannot carry on any self-care, totally confined to bed or chair”; 5, “Dead”. Each cancer-specific dataset also included a global measure of patient-perceived health state: either the EuroQol 5 Dimension 0–100 visual analogue scale (VAS) (“Please mark an X on the scale to indicate how your health is TODAY”) or a 0–10 scale with wording such as “We would like you to indicate on this scale how good or bad your own health is today”. For the purposes of this study, all respondents’ scores were standardised to a 0–10 scale by taking any 0–100 VAS responses and splitting the scores into categorical variables of 10 points each.

Demographic and disease-related characteristics of study participants were also collected. These included age, sex and diagnosis/cancer site. We also totalled the number of cancer treatment side effects collected by asking the physicians the question “What side effects is this patient experiencing on their current drug treatment?” on the PRF. However, physician-reported data on treatment side effects were not available for participants with gastric cancer, NSCLC and PC; for this reason, responses to the above question were only included to inform the assessment of the relationship between other PROs and burden.

### Statistical analyses

For all statistical tests, *p* values of 0.05 were considered significant. No correction was applied for multiple comparisons. All statistical analyses were conducted in SAS version 9.4 [27].

#### Item distributions

To understand the distribution of item responses on GP5, the percentage of patients selecting each response option was displayed on stacked bar charts and tabulated. Trends of item response use were assessed between cancer sites and, separately, between countries. This allowed for visual inspection of differences. Cancer sites were included for this presentation only when there were GP5 data from more than 100 participants.

#### Convergent validity

Convergent validity was assessed through correlations with a measure hypothesised to be closely related to the construct under investigation, patient self-reported overall health. Polyserial correlation coefficients were used to assess the relationship between GP5 and global health. This analysis was then repeated for each country. Moderate correlations (~ *r* = 0.30–50) were expected [28].

#### Known groups validity

Known groups validity was evaluated through the understanding that patients who have a more severe expression of the disease, as rated by an external marker, may be more likely to report a higher level of side effect bother. We first assessed known groups validity by estimating mean FACT-G total scores within each GP5 response category, stratified by country and by cancer type. For these analyses, we hypothesised that FACT-G total scores would decrease monotonically with increasing magnitude of side effect bother. Next, we dichotomised GP5 responses as high side effect bother (“Somewhat”, “Quite a bit”, “Very much”) and low side effect bother (“Not at all” or “A little bit”). We then conducted a series of chi-square tests to test the following known groups hypotheses for the dichotomised GP5. First, we compared dichotomised GP5 responses across groups of patients reporting any cancer-related symptoms (vs. no symptoms); we hypothesised that symptomatic patients would be more likely to indicate high side effect bother (vs. low side effect bother). Then, we stratified patients both by cancer stage and ECOG PSR and compared consecutive groups of patients’ dichotomised GP5 score. For these tests, we hypothesised that patients in higher stages of cancer and with higher ECOG PSR would be more likely to have higher side effect bother reported on GP5. These analyses were conducted on the overall sample only.

#### Differential item functioning

In order to determine whether GP5 functioned the same way for all patients both across cancer sites and across countries, DIF was examined. DIF was tested by estimating deviation between cancer sites and countries using the dichotomised GP5 groupings (“Not at all” and “A little bit” vs. “Somewhat” “Quite a bit” and “Very much”). Participants were ranked by FACT-G total score and divided into 5 severity strata for the purposes of conducting a Mantel–Haenszel test. The Cochran–Mantel–Haenszel statistic was computed and converted to a standard metric, the delta scale, where the resulting value can be judged in terms of its magnitude: < 1, negligible difference; 1 ≤ *χ* < 1.5, moderate difference; ≥ 1.5, large difference [29].

Due to the head-to-head nature of this assessment, where each cancer site was tested against each other cancer site and each country was tested against each other country, single occurrences of large DIF between two cancer sites or two countries were not highlighted specifically. Instead, patterns of large DIF occurring for specific countries or specific cancer sites were reported. Cancer sites were included for this analysis only when there were GP5 data from more than 100 participants.

## Results

### Participants

In total, 6755 cancer patients were included in this study. The highest proportions of participants came from Germany (25% of total, *n* = 1663), France (23%, *n* = 1562) and Spain (16%, *n* = 1068). In addition, a large sample of participants came from the USA (15%, *n* = 1045). The most common cancer sites among participants were breast (21% of total, *n* = 1407), NSCLC (16%, *n* = 1078), melanoma (14%, *n* = 913) and gastric (12%, *n* = 802). The proportion of cancer sites sampled across countries varied substantially in some cases (e.g. breast cancer, MM) but was fairly similar in others (gastric, melanoma). The mean age was very similar across countries, ranging from 63 to 65, and the proportions of female patients across countries ranged from 39% (UK) to 57% (Italy). Within each country, the largest proportions of patients had an ECOG PSR of 1 (ambulatory and working), with percentages ranging from 41 (Italy) to 60% (UK). The mean number of cancer side effects reported by a patient’s physician varied across countries from 4 (Germany) to 6 (France). Finally, the average rated global health (out of 10) ranged between 5.8 (France, Italy, Spain) and 7.0 (UK) (Table 1).

**Table 1:** Study participant characteristics (*N* = 6755)

	**France (*n* = 1562)**	Germany **(*n* = 1663)**	Italy** (*n* = 856)**	Spain** (*n* = 1068)**	UK **(*n* = 561)**	USA** (*n* = 1045)**
Age, mean (SD)	64 (12)	63 (11)	63 (10)	63 (11)	65 (11)	64 (11)
Female patients, *n* (%^a^)	770 (49)	807 (49)	469 (57)	501 (47)	219 (39)	586 (56)
Disease stage, *n* (%^a^)						
2	56 (17)	40 (12)	24 (7)	20 (6)	30 (9)	165 (49)
3	178 (13)	444 (33)	244 (18)	177 (13)	44 (3)	279 (20)
4	968 (24)	939 (23)	531 (13)	740 (18)	427 (11)	452 (11)
Tumour site, *n* (%^a^)						
Breast	271 (17)	246 (15)	272 (32)	278 (26)	104 (19)	236 (23)
CLL	-	-	-	-	-	362 (35)
DLBCL	-	-	-	-	39 (7)	-
FL	228 (15)	169 (10)	82 (10)	89 (8)	23 (4)	-
Gastric	261 (17)	218 (13)	91 (11)	150 (14)	82 (15)	-
HCC	-	240 (14)	130 (15)	124 (12)	-	251 (24)
Melanoma	214 (14)	239 (14)	110 (13)	108 (10)	46 (8)	196 (19)
MM	342 (22)	185 (11)	29 (3)	113 (11)	49 (9)	-
NSCLC	246 (16)	366 (22)	142 (17)	206 (19)	118 (21)	-
PC	-	-	-	-	100 (18)	-
Current ECOG PSR, *n* (%^a^)						
0, fully active	362 (23)	412 (25)	265 (31)	244 (23)	163 (29)	292 (33)
1, ambulatory and working	729 (47)	765 (47)	344 (41)	586 (55)	337 (60)	439 (49)
2, ambulatory and not working	369 (24)	354 (22)	166 (20)	188 (18)	52 (9)	98 (11)
3, limited self-care	82 (5)	96 (6)	54 (6)	44 (4)	8 (1)	53 (6)
4, completely disabled	16 (1)	9 (0.5)	15 (2)	3 (0.3)	0 (0)	8 (1)
Number of cancer side effects, mean (SD)	6 (4)	4 (3)	5 (3)	5 (4)	5 (4)	5 (5)
Global health rating, mean (SD)	5.8 (2.1)	6.1 (2.1)	5.8 (1.9)	5.8 (2.0)	7.0 (1.6)	6.6 (2.0)

^a^%’s are percentages of the row number

*CLL*, chronic lymphocytic leukaemia

*DLBCL*, diffuse large B cell lymphoma

*ECOG PSR*, Eastern Cooperative Oncology Group Performance Status Rating

*FL*, follicular lymphoma

*HCC*, hepatocellular carcinoma

*MM*, multiple myeloma

*NSCLC*, non-small-cell lung carcinoma

*PC*, prostate cancer

*SD*, standard deviation

### Distribution of GP5

**Fig. 1:**
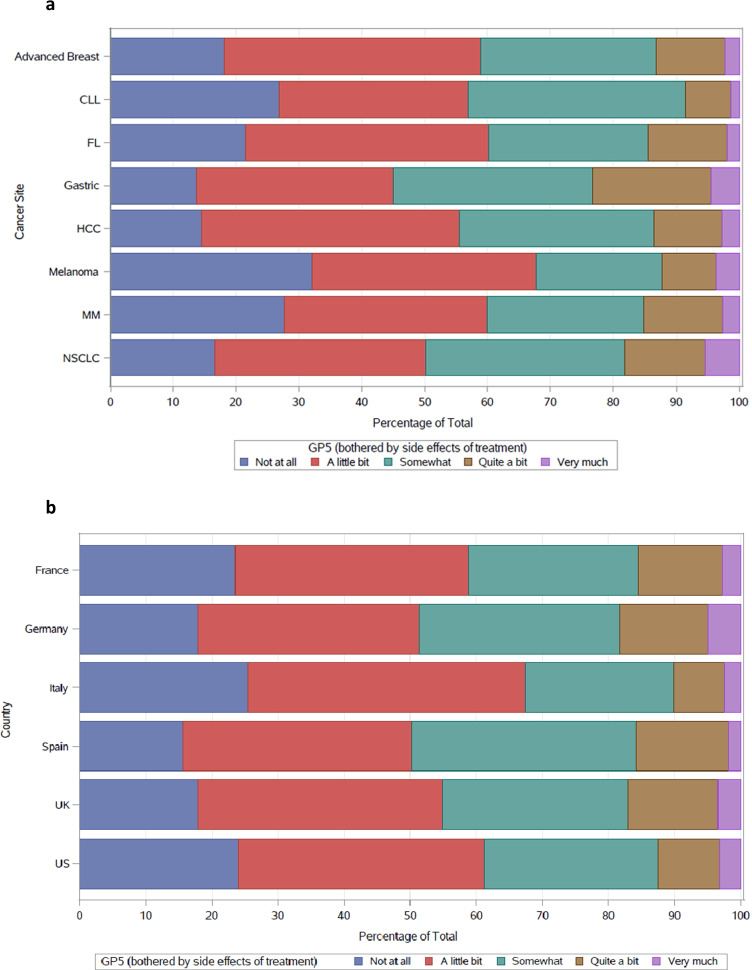
**a** GP5 responses across cancer site^a^. ^a^Diffuse large B cell lymphoma and prostate cancer are excluded from this analysis because their sample size was ≤ 100; CLL, chronic lymphocytic leukaemia; FL, follicular lymphoma; HCC, hepatocellular carcinoma; MM, multiple myeloma; NSCLC, non-small-cell lung carcinoma. **b** GP5 responses across country. UK, United Kingdom

GP5 scores were distributed similarly across most cancer sites, except for melanoma and MM. Patients with these cancers responded “Not at all” more often than patients with other types of cancers. In addition, patients with gastric cancer and NSCLC more frequently endorsed the more severe ratings than those with other cancers (Fig. 1a). The distribution of GP5 responses was similar across all countries except Italy, where the lower end of the scale (“Not at all” and “A little bit”) had proportionally more responses than other countries (Fig. 1b). Cancer sites and countries that displayed a different distribution of responses were given particular attention in the assessment of DIF.

#### Convergent validity

Overall, a moderate, negative correlation (*r*_polyserial_ =  − 0.43) between GP5 responses and global health evidenced convergent validity. These results varied across countries, with the maximum correlation between GP5 and global health observed for Germany (*r*_polyserial_ =  − 0.48) and the minimum observed for the UK (*r*_polyserial_ =  − 0.36) (Table 2). Similarly, when assessing convergent validity between GP5 responses and cancer site, moderate negative correlations were revealed for most cancer sites (Table 2). A deviation from this pattern was DLBCL, which had a weak correlation, potentially due to a small sample size (*r* = 0.17, *n* = 39).

**Table 2:** Correlations between GP5 response and overall health rating

By country		By cancer site	
Country	Correlation^a^	Cancer site	Correlation^a^
France	− 0.42	Breast	− 0.40
Germany	− 0.48	CLL	− 0.57
Italy	− 0.45	DLBCL	− 0.17
Spain	− 0.44	FL	− 0.45
UK	− 0.36	Gastric	− 0.46
USA	− 0.46	HCC	− 0.34
-	-	Melanoma	− 0.44
-	-	MM	− 0.44
-	-	NSCLC	− 0.43
-	-	Prostate	− 0.44

^a^Estimated with polyserial correlation coefficient

*CLL*, chronic lymphocytic leukaemia

*DLBCL*, diffuse large B cell lymphoma

*FL*, follicular lymphoma

*HCC*, hepatocellular carcinoma

*MM*, multiple myeloma

*NSCLC*, non-small-cell lung carcinoma

*UK*, United Kingdom

*USA*, United States of America

#### Known groups validity

**Fig. 2:**
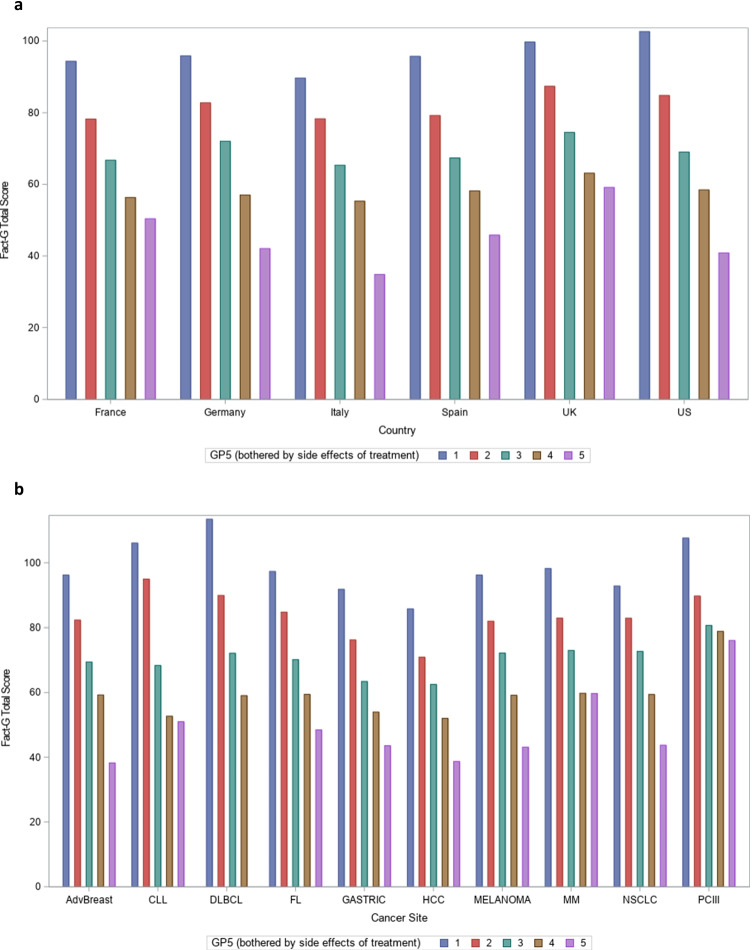
**a** FACT-G total scores by GP5 response and country. For all within country comparisons, *p* < 0.0001; FACT-G, Functional Assessment of Cancer Therapy-General; UK, United Kingdom. **b** FACT-G total scores by GP5 response and cancer site. For all within cancer site comparisons, *p* < 0.0001; CLL, chronic lymphocytic leukaemia; DLBCL, diffuse large B cell lymphoma; FACT-G, Functional Assessment of Cancer Therapy-General; FL, follicular lymphoma; HCC, hepatocellular carcinoma; MM, multiple myeloma; NSCLC, non-small-cell lung carcinoma; PC, prostate cancer

Examining FACT-G total scores by GP5 score, the expected pattern of scores was observed for the overall sample (*F*(4,5517) = 747.67, *p* < 0.001) and within each country (all *p* < 0.001) and each cancer site (all *p* < 0.001) (Fig. 2a and b). In each instance, the highest mean FACT-G scores were observed for patients responding “Not at all” on GP5. Then, scores decreased monotonically with each response category, with the lowest mean FACT-G scores observed for patients responding “Very Much” on GP5.

Next, we found evidence for each of our known groups validity hypotheses around ECOG PSR and cancer stage. For these analyses, a sensitivity analysis dichotomised responses as “Not at all” or “A little bit” (low side effect bother) vs. “Somewhat”, “Quite a bit” or “Very much” (high side effect bother). Sensitivity analysis with the dichotomised cut point between “Somewhat” and “Quite a bit” was very similar, although the stage of disease difference was no longer significant. First, a significantly higher proportion of patients who reported high side effect bother on GP5 was symptomatic rather than asymptomatic (80% vs. 64%, *X*^2^(1, *n* = 3658) = 95.64, *p* < 0.001) (Table 3). Similarly, we found that higher proportions of patients in the more advanced cancer stages tended to report higher side effect bother (*X*^2^(2, *n* = 5669) = 20.72, *p* < 0.001). Finally, as hypothesised, the proportion of patients reporting high side effect bother increased as ECOG PSR increased (*X*^2^(4, *N* = 6441) = 337.95, *p* < 0.001). Descriptively, these findings indicate a plateau in the proportion of patients reporting high side effect bother on GP5 at the later cancer stage and ECOG PSR.

**Table 3:** Known groups validity: dichotomised GP5 response by whether patients reported cancer-related symptoms

	GP5 high side effect bother	GP5 low side effect bother	*p* value
Reported cancer symptoms, *n* (%^a^)			< 0.001
Symptomatic	2161 (80)	542 (20)	
Asymptomatic	613 (64)	342 (36)	
Cancer stage, *n* (%^a^)			< 0.001
2	233 (70)	98 (30)	
3	1094 (81)	251 (19)	
4	3205 (80)	788 (20)	
Current ECOG PSR, *n* (%^a^)			< 0.001
0	1095 (64)	608 (36)	
1	2605 (83)	548 (17)	
2	1053 (88)	148 (12)	
3	303 (91)	30 (9)	
4	47 (92)	4 (8)	

^a^%’s are percentages of the row number

*ECOG PSR*, Eastern Cooperative Oncology Group Performance Status Rating

#### Differential item functioning

Standardised delta scale Cochran–Mantel–Haenszel statistics calculated across cancer sites showed large DIF (≥ 1.5) between some sites (e.g. HCC, melanoma, CLL); however, there was no overall and consistent pattern of large DIF for GP5 across cancer sites (Table 4). When assessing DIF for each country, a strong and consistent pattern of DIF was found for GP5 for Italy (Table 5).

**Table 4:**
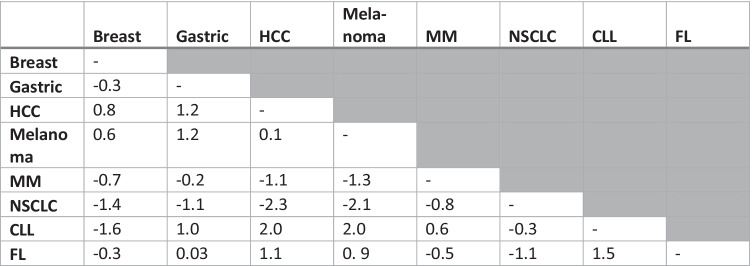
Differential item functioning: Cochran–Mantel–Haenszel statistics by cancer site^a^

^a^Diffuse large B cell lymphoma and prostate cancer are excluded from this analysis because their sample size was ≤ 100

*CLL*, chronic lymphocytic leukaemia

*FL*, follicular lymphoma

*HCC*, hepatocellular carcinoma

*MM*, multiple myeloma

*NSCLC*, non-small-cell lung carcinoma

**Table 5:**
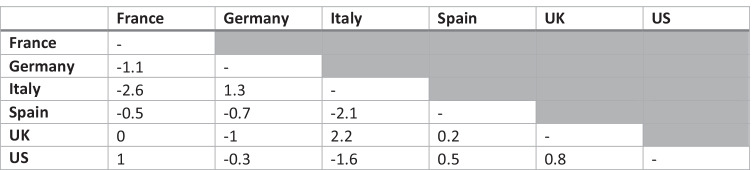
Differential item functioning: Cochran–Mantel–Haenszel statistics by country

*UK*, United Kingdom

*US*, United States

## Discussion

The use of PROs in oncology plays an increasingly important role in regulatory review of cancer treatments [13]. As new treatments emerge that extend the lives of cancer patients, the ability to assess tolerability using PROs has gained attention [12]. Though multiple options for patient-reported tolerability assessment exist, the best approach has not yet been determined. This study generated evidence for the validity of a general, single-item assessment of tolerability from the FACT system of PROs: GP5.

Results of this study indicate comparability of GP5’s performance across cancer types and in multiple countries. We stratified convergent validity analyses by both country and cancer type and found similar results across these groups. For example, the correlation between GP5 and overall health was similar whether the overall sample or each country individually was examined. In addition, the mean FACT-G scores exhibited the expected monotonic decrease with more severe GP5 responses within each country and cancer sites. In addition, with the exception of Italy, our analyses revealed very little evidence of DIF between countries or cancer sites. It is difficult from the present dataset to understand further why patients in Italy used this item differently from patients in other countries. Further work should assess this effect and perhaps revisit the linguistic validation of the Italian version of the GP5 item to see if there are any nuances which could be driving this effect. Overall, however, these results support GP5’s flexibility and appropriateness for use in multiple research or clinical oncology settings. Given that GP5 is drawn from the FACT-G, a commonly used PRO measure that is “generic” to cancer type and has evidenced validity in a diverse range of oncology populations [16, 30–33], it is not surprising that GP5 would perform well as a generic indicator as well.

Regarding cancer stage and ECOG PSR, GP5 best distinguished between the individuals with least advanced disease or lowest impairment and the next highest levels of disease progression or impairment. That is, the largest differences in proportions of patients with high vs. low side effect bother were observed for cancer stage 2 vs. 3 instead of stage 3 vs. 4. Likewise, the largest differences were observed between ECOG PSR 1 vs. 2 instead of the higher statuses. This result is likely an artefact of dichotomising between “Not at all” and “A little bit” vs. “Somewhat”, “Quite a bit” and “Very much”. This dichotomy of GP5 has been used previously and was significantly associated with probability of treatment discontinuation in a trial with breast cancer patients [34]. While this approach to operationalising GP5 has benefits, like easing interpretation of results, it may also mask variation in side effect bother among more severely impaired patients.

One potentially important application of GP5 is its use as a patient-reported measure of cancer drug tolerability in trials and real-world studies. In April 2017, through their PRO Consortium, the US Food and Drug Administration and the Critical Path Institute hosted a public workshop focusing on the use of PROs to measure tolerability in cancer trials [12]. This workshop focused most on the US National Cancer Institute’s (NCI) Patient-Reported Outcome version of the Common Terminology Criteria for Adverse Events (PRO-CTCAE) [35]. The PRO-CTCAE is comprised of 124 items covering 78 unique toxicities related to cancer therapies in terms of frequency, severity, interference, presence/absence and amount. The PRO-CTCAE items have demonstrated attractive measurement properties such as test–retest reliability and construct validity [36]. Each PRO-CTCAE item targets a specific toxicity, but, to date, there is not a recommended approach to combining responses to items to quantify overall AE burden. Given its level of generality, GP5 may be a useful complement to the PRO-CTCAE in cancer trials, helping to evaluate the overall burden of treatment-related toxicity. Since it is only a single item, it does not represent much additional assessment burden. In addition, like the PRO-CTCAE, GP5 is assessed with a context of the past 7 days, making its responses easily relatable to the PRO-CTCAE.

This study has some limitations to consider while interpreting its results. Though a very diverse pool of patients representing multiple cancer sites and European countries was used, this sample may not be representative of particular populations of cancer patients, for example, the overall UK cancer patient population, or the European HCC patient population. To confirm our results, additional study within these populations would be useful. Further to this, although a broad range of cancer sites were included, it is not necessarily representative of all cancer sites, and care should be taken when applying the GP5 item in any specific form of the disease. In addition, this study represents a secondary analysis of multiple datasets and a single time point. Though novel secondary uses of data are important to reduce research waste, there are also limitations, such as varying sample sizes for key comparison groups that may hinder hypothesis testing. Since studies are rarely designed to explore the measurement properties of PROs, secondary analyses of PRO measure data are common. Nonetheless, additional evidence would help to examine further the validity of GP5, and longitudinal data could be used to assess the validity of this item over time.

In conclusion, a single item from the FACT-G, “I am bothered by side effects of treatment” (GP5), exhibited validity among a very diverse range of cancer patients across several European countries and the USA. Little is known about a patient’s ability to differentiate cancer symptoms from treatment side effects. This can even be challenging for clinical experts [37–42]. Nevertheless, the single side effect bother question is easily understood by patients and appears to be a good indicator of overall health and performance status. These results add new, important evidence to inform the growing interest in capturing patient-reported treatment tolerability among cancer patients. As a single item, GP5 can be easily added to the protocols of real-world drug evaluation studies and trials alike without increasing assessment burden. Since such studies increasingly feature international patient populations, the results of this study help to build confidence that GP5 can be used among cancer patients from multiple countries. In addition, since GP5 performed well across multiple cancer sites, it can be used to compare treatment tolerability broadly in oncology research.

## Data Availability

All data that support the findings of this study are the intellectual property of Adelphi Real World. All requests for access should be addressed directly to Alex Rider at alex.rider@adelphigroup.com. The FACIT and all related works are owned and copyrighted by and the intellectual property of David Cella, Ph.D. Permission for use of the FACT-G questionnaire is obtained by contacting Dr. Cella at information@facit.org.
